# The Role of Local Knowledge and Traditional Extraction Practices in the Management of Giant Earthworms in Brazil

**DOI:** 10.1371/journal.pone.0123913

**Published:** 2015-04-14

**Authors:** Maria Auxiliadora Drumond, Artur Queiroz Guimarães, Raquel Hosken Pereira da Silva

**Affiliations:** Departamento de Biologia Geral, Instituto de Ciências Biológicas, Universidade Federal de Minas Gerais, Belo Horizonte, Minas Gerais, Brazil; NERC Centre for Ecology & Hydrology, UNITED KINGDOM

## Abstract

The giant earthworm, *Rhinodrilus alatus* (Righi 1971), has been captured in the southeastern Brazilian Cerrado biome for approximately 80 years and used as bait for amateur fishing throughout Brazil. Local knowledge and traditional extraction practices are crucial for the establishment of management strategies for the species because, although its extraction involves conflicts and social and environmental impacts, the species is one of the major sources of income for approximately 3,000 people, especially for members of an Afro-descendant community that has approximately 2,000 inhabitants. Participatory tools, such as seasonal calendar, transect walks and participatory maps, were individually or collectively used with extractors and traders (former extractors), and 129 semi-structured and unstructured interviews were conducted with the same individuals between 2005 and 2012. The capture of *Rhinodrilus alatus* was observed in different seasons and areas of occurrence of the species in 17 municipalities, where this giant earthworm is the only species extracted for trade. All information obtained was verified by community members in 17 meetings. The extractors have an extensive knowledge of the life history, behavior, distribution, and possible impacts of climate change on the species. Different capture techniques, which have different impacts, are used during the dry and rainy seasons and are passed by the extractors through the generations. Local knowledge contributed to the establishment of agreements for the use of capture techniques that have less impact, to the expansion of scientific knowledge and the reassessment of the conservation status of *Rhinodrilus alatus*. The present study may serve as an example for management projects for other giant earthworm species in other regions of Brazil and in other countries.

## Introduction

Recognition of the knowledge, practices and beliefs that are accumulated by society from one generation to the next has gone through different stages, from its marginalization in the 1960s until its adoption as a standard in conservation projects in the 1990s [1]. It is currently accepted that the incorporation of the knowledge and perspective of people who use or depend on natural resources into the joint formulation of knowledge and decision-making generates positive results in management programs and policies [1–6]. Local knowledge can contribute to the conservation of biodiversity, protected areas management, sustainable use of natural resources, maintenance of ecosystem services, environmental impact assessment and to improve public policy strategies [2, 7–15].

Ethnozoological studies are very valuable for the knowledge of bioecology and of sociocultural links between humans and animals and, through them, is possible to identify and understand the use of wildlife for different purposes like for food, traditional medicine, trade, religion and culture [2, 16–25]. This approach also play a key role in wildlife conservation and management, contributing to the discovery of new species, new records of species, detection of invasions or extinctions [20, 26, 27], mapping of species distribution, and for identifying priority areas for conservation [28–32]. Hunting pressure and other conflicts [20, 30, 33–36], as well as temporal changes in population and community dynamics can be identified from local knowledge [26, 37–39]. Some of these data can also contribute to species conservation status assessment [30, 37–40].

However, despite this importance, not always this knowledge can be readily added in sustainable wildlife use programs [41]. In Brazil, for example, there are several legal and institutional barriers to the use of wild animals, although it is an entrenched practice throughout the country [17–20, 23–24]. Except for indigenous peoples, whose activities and traditional ways of life are guaranteed by the Brazilian Federal Constitution, the use of native animal species is banned since the 1960s, with the promulgation of the Wildlife Protection Law (Law 5,197/1967). The Environmental Crimes Law (Law 9.605/1998) also prohibits the collection, hunting and persecution of native fauna without permission. Legally, only the subsistence hunting and animal slaughter to protect agricultural activities or in cases of harmful species are not crimes. On the other hand, national policies related to fish resources, which include exploitable aquatic organisms, encourage their use (Law 11,959/2009), unlike the inflexible rules of protection to other components of the native fauna. Furthermore, although terrestrial invertebrates are legally recognized as native fauna and thus are subjected to same legal restrictions of vertebrates, they are generally used and traded without major restrictions [42, 43]. This context may explain the fact that themes like ethnoictiology and ethnoentomology represent a significant part of publications on ethnozoology in Brazil if compared to other animal groups [21]. In addition to legal aspects, other factors can interfere in the development of ethnozoological studies such as conflicts related to common use of resources or invasions in particular properties for catching animals. All these aspects are included in this study, since the conflicts related to the use of an earthworm species are intense.

Earthworms have great importance for the maintenance of ecological processes, like organic matter decomposition and nutrient cycling [44–46]. They are directly used by humans, mainly for humus production [46–48] and as fish bait [45, 49, 50]. The medicinal use of earthworms is widely known and it was already reported to treat, for example, asphyxiation, relief of rheumatic pains, fever, postpartum weakness, smallpox, to reduce bladder stones, to cure yellow skin in patients with jaundice, to reduce hair loss [50–52], and many other pharmacological applications are being tested [52–56]. In Latin America, earthworms are important in the diet of some indigenous peoples of the Brazilian Amazon and are recommended by local healers for treating malaria or anaemia, or for women after parturition [57, 58]. This is also the case for some Venezuelan Indian tribes [58–60]. Despite the ecological importance and the use of these organisms by humans, they are not frequently cited in ethnozoology publications in Brazil [2, 21].

The giant earthworm, *Rhinodrilus alatus* Righi (1971), popularly known as “minhocuçu”, is one of the 54 species of large earthworms that are known to occur in Brazil and is endemic to the Cerrado biome [45]. This species, used as fish bait for about 80 years, can reach 1.30m in length and has been found only in the State of Minas Gerais in southeastern Brazil [61]. Recreational fishing with *R*. *alatus* is held in the midwest, southeast, north and northeast of Brazil, especially in the Pantanal region. Large freshwater fishes such as “surubim” (*Pseudoplatystoma corruscans*), “mandi” (*Pimelodus maculatus)*, “matrinchã” (*Brycon* sp.), “piraputanga” (*Brycon* sp.), “cachara” (*Pseudoplathystoma fasciatum)*, “barbado” (*Pinirampus pinirampu)* and “pirarara” (*Phractocephalus hemeliopterus)* are among the species caught using “minhocuçu” as bait. To prepare the bait for fishing these large species, a whole earthworm is compressed in a hook (5–7cm) with a hard stainless steel leader (>20cm), and for catching smaller species earthworms are cut into smaller pieces.

Approximately 3,000 people are involved in *R*. *alatus* extraction and trade, and this activity may be their main source of employment and income. This is especially true for a traditional Afro-descendant community (“quilombola”) that is located in the municipality of Paraopeba, in central Minas Gerais [49,61]. The extraction activities involve conflicts such as invasion of private property and of a Federal Protected Area (Paraopeba National Forest) and the non-authorized use of wildlife, which cause giant earthworm extraction to be an environmental crime under Brazilian law. Despite the scarcity of information about the species, *R*. *alatus* was designated as an endangered species in the State of Minas Gerais (Council on Environmental Policy Normative Deliberation 41/1995) in 1995 and in Brazil (Ministry of Environment Normative Instruction 03/2003) in 2003. Its inclusion on these endangered species lists was based on intensive collection practices, habitat destruction and on its restricted distribution to only two municipalities [62]. *R*. *alatus’* inclusion on these lists did not eliminate its use but added more legal obstacles, increasing the penalties prescribed by Law 9.605/1998.

In order to assess the viability of *R*. *alatus* management and try to mitigate the conflicts associated to its use, in 2004 was created the “Minhocuçu Project”, developed through partnerships between public and private institutions and local stakeholders. The limited knowledge about the biology and ecology of *R*. *alatus* at the beginning of the project was one of the bottlenecks for the development of a conservation and management proposal. Studies of giant earthworms are more difficult to develop than studies of smaller earthworms, mainly because giant earthworms are hard to collect. However, this issue is minimized when the study incorporates the knowledge and practices that have accumulated over nearly one century of extraction and use of giant earthworms by the local community.

Whereas ethnozoological studies may contribute to development of conservation strategies, this work provides local knowledge about *R*. *alatus* and also analyzes how this information can be incorporated into a management program for the species. The conceptual and methodological basis of "Minhocuçu Project" is the adaptive management, which involves a constant learning process that links scientific research to action, takes the complexity of systems and uncertainties into consideration, and can be modified and improved through action [63–67]. This is especially important when the need for change is urgent, as the socioecological context of earthworms’ traditional use presented in this manuscript.

## Materials and Methods

### Study area

The study area is located in the Cerrado biodiversity hotspot [68], a biome that covers approximately 22% of Brazil [69]. *Rhinodrilus alatus* occurs in 17 municipalities in the central region of the State of Minas Gerais (N 17.600141, S 19.613570; E 43.736822, W 45.361379), where there are different types of Cerrado vegetation physiognomies, pastures and *Eucalyptus* plantations [61]. The 17 municipalities within which the occurrence of *R*. *alatus* was confirmed were visited by the research team, which included biologists and local experts. The team collected the animals in areas that had been previously identified by the extractors and confirmed the species’ presence. Most of this work was conducted in the municipalities that have the highest concentration of extractors and traders (see below). Community meetings were held in the Pontinha Community Association, in public schools and in the Paraopeba National Forest (Floresta Nacional de Paraopeba), which is located in the municipality of Paraopeba.

### Data collection and analysis

Participatory tools, such as semi-structured interviews, participatory maps, seasonal calendars and transect walks [70–75], were used in the present study. Traders and extractors of giant earthworms who lived in the municipalities of Caetanópolis, Curvelo, Paraopeba, Pompéu, Sete Lagoas, Corinto and Três Marias participated individually or in small groups in 129 semi-structured and unstructured interviews. Some of them participated several times. Only giant earthworm traders were approached initially because they were easier to locate at their points of sale. The initial interviews were difficult because the use of giant earthworms is illegal. Data collection was only possible after building trust relationships with the interviewees, which occurred during the first year of work.

The permissions for entry into private properties were verbal and permission to work with wild fauna was given by the Authorization and Information System in Biodiversity (SisBio), from Chico Mendes Institute for Biodiversity Conservation (ICMBio), Ministry of the Environment (MMA) (authorization number: 30164–4). Regardless of the project, there are informal agreements between extractors and landowners, who lease part of their property for the extraction, even though this practice is still illegal. The contacts with the land owners were made through the extractor or trader who organized the extraction events. The present study was registered with the Board of Ethics in Research (process number 03329412.1.0000.5149) to ensure the integrity and rights of the individuals who voluntarily agreed to participate in the study.

The participatory maps were used with six traders to gather information about the extraction sites and to increase knowledge of the distribution of the species. The different stages of the annual life cycle of giant earthworms, which were initially described in interviews, were subsequently visualized on a seasonal calendar that was developed by two of the traders. The research team monitored the extraction process in 17 municipalities, in different areas of the Cerrado and in pastures and *Eucalyptus* plantations. The team recorded the stages of the life cycle of *R*. *alatus* and the traditional techniques that were used in their extraction in both the dry and rainy seasons.

The resulting information was summarized and shared with the local community in a planning workshop that was convened to discuss a management agreement. Eighty-three participants, including extractors, traders, landowners and environmental agencies, attended the planning workshop.

The use of different participatory tools and several information sources supported a greater understanding of the information based on the triangulation strategy [73, 76]. The local knowledge of the extractors was recorded in a documentary video that was discussed with traders and extractors in a meeting with 12 participants and in a booklet that was discussed with traders and extractors in a meeting with 24 participants.

All interviews were recorded and entered for use with *ATLAS*.*ti* 7 software (Scientific Software Development GmbH, Berlin, Germany). Recordings, field notes and photographs were archived at the Laboratory of Socio-Ecological Systems of the Federal University of Minas Gerais (Universidade Federal de Minas Gerais—UFMG).

## Results and Discussion

### Local knowledge of the life history of giant earthworms

**Fig 1:**
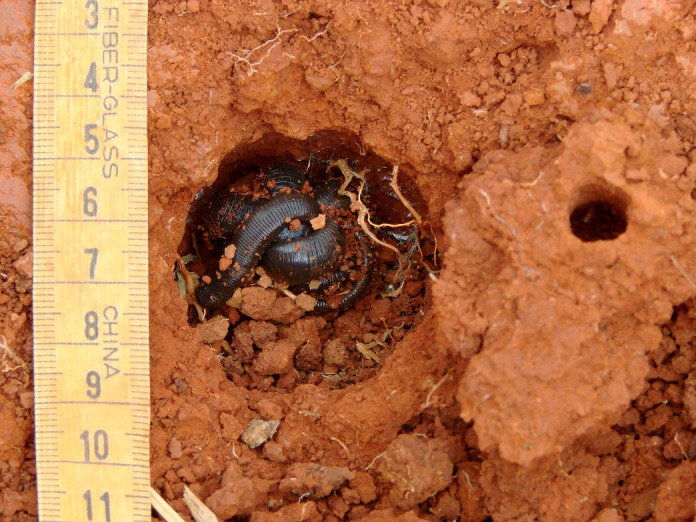
*Rhinodrilus alatus* in its aestivation chamber during the dry season.

**Fig 2:**
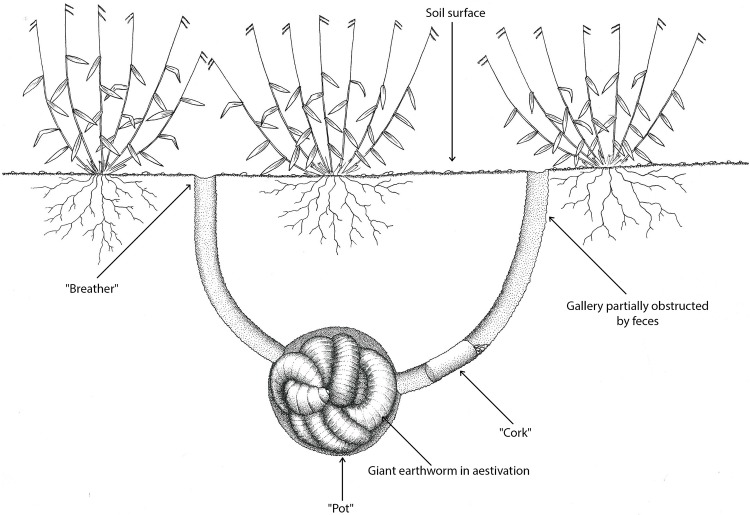
Scheme of “minhocuçu” in its aestivation chamber. During the construction of the “pot” the giant earthworms build two galleries. One of them may be obstructed by the last feces that was expelled before aestivation (“cork”) and the second gallery (with a “breather”) connects the aestivation chamber to the ground surface. Created by Samuel Hosken.

**Fig 3:**
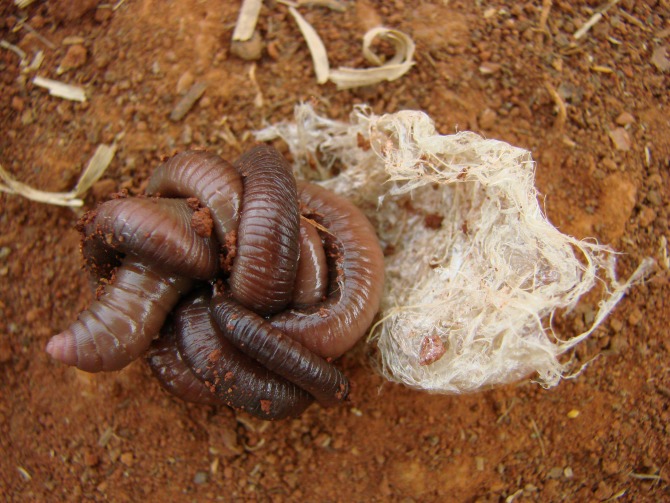
*Rhinodrilus alatus* and its “silk”. During the drier months, giant earthworms excrete mucus (“silk”, “wool”, “web” or “cloth”) that covers the bottom of the aestivation chamber which retains water and allows the worms to stay moist. The coiled worm has about 5 cm diameter.

**Fig 4:**
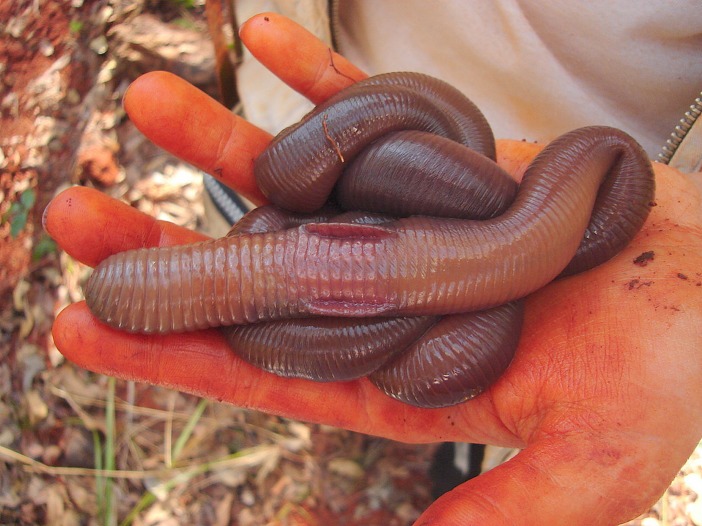
Mature specimens of *Rhinodrilus alatus* with laterally projected clitellum.

Giant earthworms stay coiled in an aestivation chamber (“pot”) for six to seven months of the year (Fig 1). The stimulus to initiate aestivation is the beginning of the dry season, and aestivation ends with the beginning of the rainy season. During the construction of the “pot”, in January and February, the giant earthworms build two galleries, an activity the extractors call “giant earthworms in corridor”. One of the galleries may be obstructed by the last feces that was expelled before aestivation (“cork”), which may serve as protection against desiccation and ant attacks. The second gallery (“breather”) is used during the movement of the animals from the aestivation chamber to the ground surface to absorb dew or rain water (Fig 2). Typically, only one giant earthworm is found in the “pot”, but in some cases, when there is a confluence of galleries at the time of chamber construction, two or three individuals may be found in one “pot”. During the drier months, giant earthworms excrete mucus (“silk”, “wool”, “web” or “cloth”) that covers the bottom of the chamber, retains water and allows the worms to stay moist (Fig 3). At the beginning of the rainy season, the giant earthworms ingest the “silk” before leaving the chamber, and the mature specimens prepare to copulate (“to become male”) and laterally project the clitellum (“dewlap”) (Fig 4). According to the reports, “the giant earthworm moves a lot and does not stay in a small area”. The extractors attribute the difficulty of rearing *R*. *alatus* in captivity to their movement over long distances during the rainy season. The extractors also believe that captive rearing is difficult because giant earthworms should not be placed in a soil type that is different from that in which it was initially found because they are very sensitive to changes, especially chemical changes.

At the rainy season, rainfall over two or three consecutive days induces giant earthworms to reproduce. They are hermaphroditic and copulate once a year, staying “head to head” during copulation and it occurs just below the soil surface. It is possible to find giant earthworms during copulation through a “crack” that is formed in the ground. During the reproductive period, the ventral surface of the giant earthworm becomes rougher (“sandpaper”) to make it easier for the individuals to copulate.

Giant earthworms “break” when handled during the rainy season. Mechanically-stimulated autotomy is an adaptive strategy of several groups of animals [77–81]. According to a review by Maginnis [82], most studies that evaluate the benefits of autotomy relate this strategy to escape from predators. *R*. *alatus* individuals sever themselves only during the breeding and foraging seasons, when they are more active and move around on the soil surface, which makes them more vulnerable to predators. Autotomy and foraging (resulting in a full digestive system) cause the giant earthworm to lose commercial value, and the demand for *R*. *alatus* decreases due to its unattractive characteristics as live bait during this season of the year.

**Fig 5:**
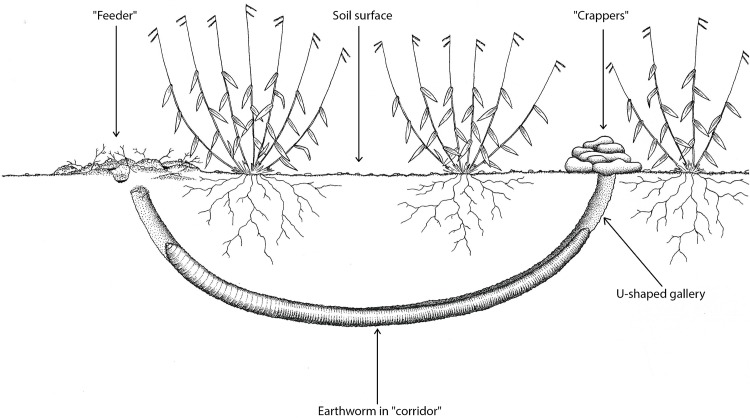
Scheme of *Rhinodrilus alatus* in “corridor”. Each individual builds a U-shaped gallery (“corridor”) after copulation and leaves traces in places where it eats (“feeders”) and where it defecates (“crappers”). Created by Samuel Hosken.

Each individual builds a U-shaped gallery (“corridor” or “channel”) after copulation and leaves traces in places where it eats (“feeders”) and where it defecates (“crappers”) (Fig 5). The cocoon, which is traditionally known as the “egg”, usually has two or three offspring and is deposited in a shallower and smaller chamber (“small pot”). The cocoon is oval (“little gourd”) and is affixed to the top of the chamber. The parent worm stays close to the cocoon, and it is difficult to find them during this time because they stop feeding and do not leave tracks on the surface. The extractors estimate that sexual maturity is reached when the giant earthworms are two to four years old, but they cannot estimate the longevity of these animals. The number of cocoons that are produced annually by each specimen is also unknown. Some extractors believe that only one cocoon is produced in each rainy season, but others report that production depends on the duration of rainfall events and that more than one cocoon can be produced if the rainy season is long. This issue needs further investigation, which may only be possible when captive breeding experiments are successful.

Some of the items in the agreement that was established among stakeholders were based on local knowledge of the life history of giant earthworms. Examples include prohibiting collection during the breeding season, avoiding extraction of immature specimens and, given the time that is required for the species to reach sexual maturity, establishing rotation of extraction areas. These measures are intended to minimize the impact of extraction on giant earthworm populations, and most extractors and traders in the study area have complied with them.

### Geographic distribution

The occurrence of *R*. *alatus* was documented in 17 municipalities by information that was gathered in participatory maps, interviews and transect walks [61]. The species shows resilience to changes in land use because it also occurs in Cerrado areas that have been converted to pastures and eucalyptus plantations. This new information about areas of occurrence is essential for reassessment of the risk of extinction of the species, according to the criteria of the International Union for Conservation of Nature. The *R*. *alatus* risk of extinction was changed from Endangered (EN) to Least Concern (LC) [83]. One of the positive impacts of the reassessment of the conservation status of *R*. *alatus* is that, despite the difficulties and limitations, it is possible to implement a plan for managing the species in the wild as long as it is approved by the public agencies that are responsible for wildlife management in Brazil and as long as extraction activities are regulated.

Due to the extensive area of occurrence and extraction of giant earthworms, the extractors and traders themselves are aware that the implementation of management programs should be at a regional rather than at a local level, even though they recognize that this broader scope makes management initiatives more difficult and institutionally more complex.

Although *R*. *alatus* occurs in disturbed areas, such as pastures and eucalyptus plantations, it only stays in these areas when pesticides are not applied because they are sensitive to these substances. Additionally, some eucalyptus species, such as *Corymbia citriodora* (Hook.) K.D. Hill & L.A.S. Johnson, produce secondary compounds [84] that seem to be toxic to giant earthworms. Giant earthworms were not found in areas, such as the Paraopeba National Forest, that have a high density of this eucalyptus species.

Information about potential factors that determine the occurrence of giant earthworms is crucial for their management and allows identification of priority areas for their conservation and areas that are most suitable for their extraction. This information also helps to identify land use and soil management practices that should be avoided in extraction areas.

### Traditional methods of extraction of *R*. *alatus*

Strategies for capturing giant earthworms vary substantially between the dry and rainy seasons and depend on the ethnozoological knowledge of the extractors, which is passed down through the generations. The extractors are local specialists and easily detect traces of the presence of giant earthworms at different stages of their annual cycle.

**Fig 6:**
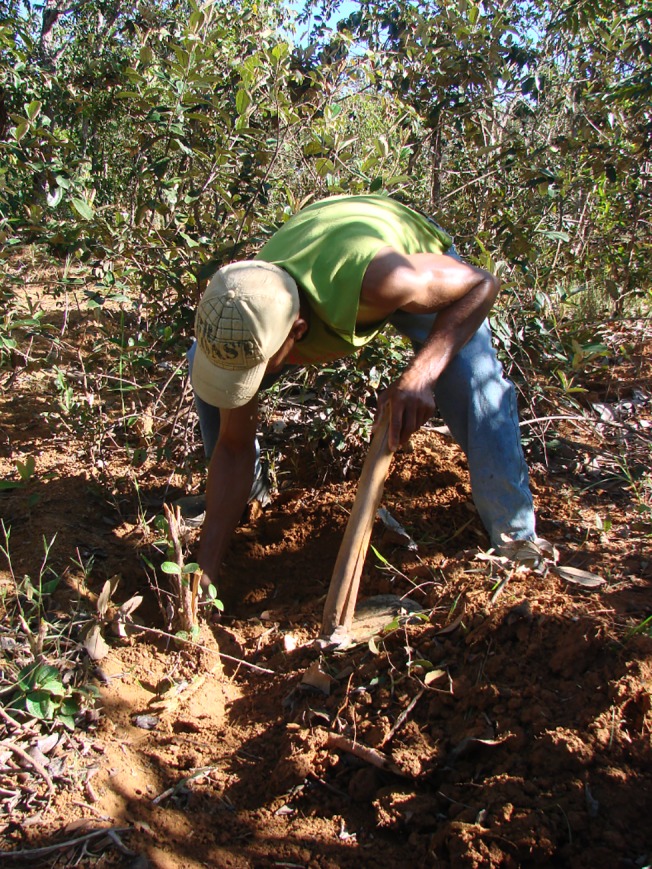
Extraction during the dry season. Traditional giant earthworm extractor using a hoe to excavation.

In the dry season, the giant earthworms can be found by the last feces (“yellows”) that are deposited before an individual begins to aestivate. These last feces clean out the earthworm’s digestive system. Giant earthworm feces are mineralized and therefore remain compact on the soil surface, where they can be seen. The extractor analyzes these traces carefully, assessing their age by feces color and moisture content. If the feces are recent, the extractor removes litter and starts digging to locate the underground gallery that leads to the aestivation chamber. Excavation is done with a hoe that is made exclusively for this activity, which has a handle that is approximately 80 cm long and a blade that is molded from a cast iron plate taken from a plow blade (Fig 6). The blade is sharpened by the extractor to facilitate the work in the extremely hard soil of the dry season.

To avoid damaging the worm, some extractors confirm its presence in the aestivation chamber by introducing a flexible plant stem (from grass or another type of plant) from the gallery into the chamber. The extractor withdraws the stem and checks to see if it is wet, which would signal the presence of a worm. Extractors also use other techniques, such as blowing into the gallery or gently touching the outside of the chamber, each of which produces a different sound when the chamber is occupied or empty. When a giant earthworm is detected, the care taken during excavation is redoubled to prevent injury to the animal during capture. The process is repeated with a search for new traces, excavation and extraction.

The route that is followed by the extractor varies with the locations of traces and occurrence of the animals. An extractor captures an average of 5.6 ± 3 specimens in an hour [61], although this number may vary according to the ability of the extractor and the abundance of giant earthworms at the extraction site. Sometimes, the extractor uses fire to clean the extraction area to more easily detect the feces of giant earthworms and their extraction sites. This practice is a key factor in increasing conflicts between extractors and landowners, makes the giant earthworms captured more fragile because of their sensitivity to ashes, and compromises the remaining population that is not captured.

The extracted giant earthworms are initially placed in fabric bags. They are transferred at the points of sale into clay pots that are especially made by women of the region to accommodate the worms. Fishermen prefer giant earthworms that are called “cured” when they have been covered by mucus during the dry season because they are more resistant and easier to transport.

The aestivation chambers are found at an average soil depth of 25.8 ± 6.8 cm [61]. There is therefore considerable soil disturbance during extraction, especially when the chambers are deeper. This extensive soil disturbance makes it difficult to locate other giant earthworms at the collection site because their traces can be covered by the disturbed soil. This prevents the area from being depleted, which favors the species’ continued existence. The presence of trees and shrubs also decreases the risk of giant earthworm depletion in a capture area. More mature trees and shrubs are not removed during extraction, and their roots provide refuge for the giant earthworms that are not captured. However, the impacts of the extraction activities on giant earthworm populations are evident in pastures, eucalyptus plantations, and Cerrado, especially when there is more than one capture effort in the same area in the same year, which the extractors call “repicking” and which occurs when the capture success of giant earthworms in other areas is low.

**Fig 7:**
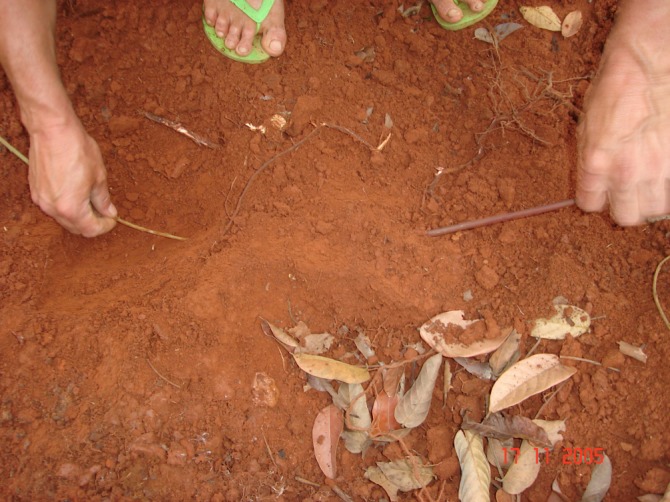
Extraction during the rainy season. Traditional extractor inserting a thin twig into the hole that is near the giant earthworm’s head, which forces the animal to retreat.

**Fig 8:**
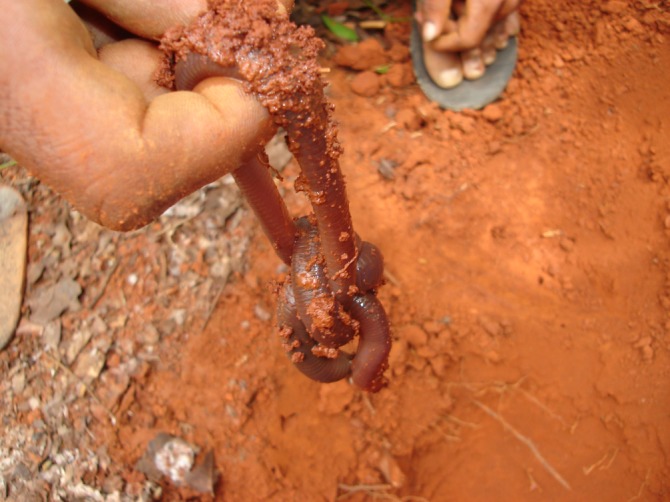
Giant earthworm just after capture.

Extraction procedures are quite different during the rainy season, when giant earthworms breed and forage. During this period, the worms are found in U-shaped galleries (“corridors”) and its depth can be very variable according to the soil moisture. The animals are located by the traces where they have fed (“feeders”) and presence of fresh feces (“crappers”). The extractor removes the litter and starts digging, clearing both openings of the gallery. The extractor then carefully inserts a thin twig into the hole that is near the giant earthworm’s head, which forces the animal to retreat (Fig 7). This causes the caudal extremity to protrude out of the other hole, and the worm is then captured (Fig 8). There is less extraction activity during the rainy season because it coincides with a period during which boat fishing is prohibited in Brazil, which decreases the demand for bait. The decreased demand and the loss of commercial value of giant earthworms during this period due to autotomy and a full digestive system contributed to the implementation of a capture prohibition during the breeding period of the species.

There are reports of annelids capture using manual collection [45,60], detergent, acetic acid, pitfall traps [45], diluted formalin [85, 86], mustard [87], potassium permanganate [88], and crabs as bait [89]. However, there are few records of traditional extraction methods of large oligochaetes. In the United States, several families residing in and around Florida’s Apalachicola National Forest have survived on the capture of large *Diplocardia mississippiensis* earthworms through a technique called ‘worm grunting’. A wooden stake is driven into the ground and scraped with a flat metal object. In response to the vibrations, worms emerge to the surface, where thousands can be collected in a few hours [90, 91]. However, an annual permit for extraction is now required, and methods involving the generation of vibrations are forbidden [90] because of the large-scale use of this technique and consequent over-exploitation.

The number of people who are engaged in the extraction of *R*. *alatus* in Brazil and the volume of sales are very large [49, 61]. However, unlike the technique that was used in the United States, extraction of *R*. *alatus* in Brazil is slower, requires more physical effort and capture success is lower. The impacts are nonetheless severe. The difficulty of capturing giant earthworms by individuals who lack tradition or family history also reduces the possibility of expanding the activity to other areas of occurrence of *R*. *alatus*. Extraction and trading of other species of large earthworms were recorded in other Brazilian regions, such as northeast of Minas Gerais and States of São Paulo, Goiás and Mato Grosso [45, 92]. However, the ecological information about these species and socioeconomic data about their use are still very scarce. The small number of oligochaetes experts in Brazil, especially taxonomists [45], limits the knowledge of these animals, even widely used species, as the “Salinas Minhocuçu”, a new species of *Rhinodrilus* sp. sold for over ten years, according to local traders. However, an increase in the number of experts in this group do not necessarily will improve the level of knowledge about the ecology of large earthworms, if the scientists do not take in account the local knowledge and tradicional practices of capture, since species studies in its habitat is very difficult. The establishment of management strategies requires therefore, the real participation of local stakeholders. The continuity of the extraction of any species without appropriate management can lead to population collapses, as occurred with *Glossoscolex paulistus* in the State of São Paulo [45].

### Effect of climatic variation on the behavior and population of *R*. *alatus*

The length of the different stages of the giant earthworms annual cycle varies with the duration of the rainy and dry seasons and with the intensity and consistency of rainfall events. Although the transition from the aestivation stage to the breeding and foraging stages is dependent on the beginning of the rainy season, interruption of the rains by a short dry period can stimulate the giant earthworms to return to the underground chamber. A new chamber may be built, which would be shallower than the previous aestivation chamber. If there is a longer period of drought after the construction of the second chamber, the mortality rate of the giant earthworms may increase because they are not able to survive in low humidity and high temperatures for a long period. However, the giant earthworms can return to foraging activity if the rains return.

Microclimatic factors and chamber depth may also affect the different stages of the giant earthworm annual cycle. For example, giant earthworms may enter aestivation earlier in Cerradão areas (Cerrado with forest formation, which have lower temperatures because they have taller trees and more shade) than in pastures. The depth of the aestivation chambers may be related to the moisture content and temperature of the soil. The deepest chambers are built in drier soils that have higher temperatures. Giant earthworms with deeper chambers may begin the reproductive period later than giant earthworms that build shallower chambers because the increase in soil moisture at greater depths occurs only when rainfall is more constant and sufficient for the deeper soil layers to remain moist. This behavior favors greater survival of giant earthworms that build deeper chambers in places where surface moisture can decrease dramatically after a short rainy season.

These adaptations to water stress may contribute to the resistance of the species in the face of climate change. However, even with these behavioral adaptations, mortality may increase during an extended drought, which would reduce giant earthworm populations and, consequently, their availability to extractors. The extractors reported that temperature increases and soil moisture decreases can reduce the populations of giant earthworms over a short time and that such changes are already a concern.

Projections of Intergovernmental Panel on Climate Change point to considerable changes in amount and distribution patterns of rainfall in South American region [93]. In Brazil, climatic extremes with severe droughts and floods are already recorded [94]. These projections and observations are crucial to know the future scenarios of *R*. *alatus* conservation and reinforce the need to increase our understanding of climate change effects in abundance, behavior and distribution of this species. The absence of long-term management strategies that consider the possibility of reduced giant earthworm stocks and areas of occurrence due to regional climatic changes [95] may make the species and its production chain sensitive to such changes [61] because environmental variation can negatively affect the intrinsic population growth rate, which is strongly influenced by survival and reproductive performance [96–98]. The impacts of climate change on fish stocks and fishing [99] also can not be ignored, since they interfere directly in demand for baits and hence in giant earthworm production chain.

## Conclusions

Local knowledge about “minhocuçus” shows, besides ethnozoological aspects, the complexity and uncertainty related to the socio-ecological system concerned to its use. Its management should incorporate assessing and monitoring risks, and actions that increase the resilience of system by responding to disturbances [63, 100, 101]. Thus, not only the species harvest should be regarded, but also changes in public policies that might affect the native fauna and fisheries policies and also the economic fluctuations that lead to changes in land use in the”minhocuçu” occurrence area or may affect the fishing activities.

Despite there being strong institutional resistance to wildlife management in Brazil due to the inflexibility of laws that govern management issues, the evidence presented in this study indicates that collaborative adaptive management of *R*. *alatus* is a viable alternative for achieving sustainability of traditional extraction activities. Despite the gaps in knowledge that still exist, particularly with respect to giant earthworm reproduction and life span, we believe that it is not possible to wait for another decade of research to recognize adaptive management as an official policy. The involvement of different stakeholders and constant monitoring of extraction and trade activities are essential for promoting knowledge and adjustments that are necessary to respond to possible changes in the environmental and socioeconomic scenarios related to this activity. As in this study, the management of other species is characterized by the complexity and discontinuous behavior in time and space, and, for this reason, it should incorporate an adaptive approach.
